# Complete Genome Sequence of *Citrobacter freundii* Myophage Mijalis

**DOI:** 10.1128/genomeA.00228-17

**Published:** 2017-08-03

**Authors:** Sruti Parvataneni, Eleni M. Mijalis, Gabriel F. Kuty Everett, Eric S. Rasche, Mei Liu, Jason J. Gill

**Affiliations:** a Center for Phage Technology, Texas A&M University, College Station, Texas, USA; b Department of Animal Science, Texas A&M University, College Station, Texas, USA

## Abstract

*Citrobacter freundii* is responsible for various opportunistic nosocomial infections. Phage therapies against *C. freundii* may prove useful in human medicine for treatment of infections caused by the ubiquitous bacteria. Here, we announce the complete genome sequence of the *C. freundii* Felix O1-like myophage Mijalis and present its features.

## GENOME ANNOUNCEMENT

*Citrobacter freundii*, a Gram-negative bacterium of the family *Enterobacteriaceae*, can cause brain abscesses and fatal infections such as bacterial meningitis (1). Because *C. freundii* is becoming increasingly resistant to antibiotics (2), the virulent phages infecting this bacterium are of interest as potential therapeutics.

Bacteriophage Mijalis was isolated from municipal wastewater in College Station, TX, in February 2015. Phage DNA was sequenced in an Illumina MiSeq 250-bp paired-end run with a 550-bp insert library at the Genomic Sequencing and Analysis Facility at the University of Texas (Austin, TX, USA). Quality controlled trimmed reads were assembled into a single contig of circular assembly at 67.5-fold coverage using SPAdes version 3.5.0. Contig completion was confirmed by PCR and sequencing of the resulting product. Genes were predicted using Glimmer3 and MetaGeneAnnotator (3, 4) and corrected using software tools available on the Center for Phage Technology (CPT) Galaxy instance (https://cpt.tamu.edu/galaxy-pub/). In keeping with the convention established by phage T4, the genome was reopened between its rIIa and rIIb homologs. Morphology was determined by negative-stain transmission electron microscopy performed at the Texas A&M University Microscopy and Imaging Center.

The 87,998-bp double-stranded DNA genome of myophage Mijalis has a coding density of 89.4% and a coding G+C content of 39.1%. Mijalis is related to the family of the classic *Salmonella-*specific typing phage Felix O1 (accession no. NC_005282) (5), with which Mijalis shares 49.8% sequence identity, as determined by EMBOSS Stretcher (6). Like Felix O1, Mijalis has significantly lower G+C content than that of its host *C. freundii* (51.6%) (7). This disparity between phage and host DNA has been found to be a common feature of related Felix O1-like phages, such as bacteriophages Moogle (accession no. NC_027293), Michonne (accession no. NC_028247), and Mordin (accession no. KT363872), which also share high sequence similarity (>85%) with Mijalis (8–10). Despite being isolated 2 years apart from different wastewater facilities, phage Mijalis and *C. freundii* phage Moogle are almost identical (differing by a single-base deletion and two SNPs), indicating that phage Mijalis represents a persistent clone that inhabits the wastewater of this region. Of the 131 predicted coding sequences in Mijalis, 44 were assigned putative functions based on BLASTp and InterProScan results (11, 12), while 87 were designated hypothetical novel or conserved. Mijalis contains 25 tRNA genes detected by ARAGORN (13), and encodes 16 predicted rho-independent terminators, compared to 22 tRNA genes and 17 terminators in Felix O1 (5).

Mijalis encodes Felix O1-like genes for DNA replication and packaging, morphogenesis, and lysis. A translational frameshift in the tape measure chaperone was identified, similar to that found in other Caudovirales phages (14). Other identified genes include those that encode for capsid, tail fiber, baseplate, and tape measure structural proteins. Based on the similarity of the Mijalis TerL to other terminases of headful packaging phages, it has been concluded that Mijalis employs pac-type headful packaging, similar to Felix O1 and T4 (15). Genes involved in lysis were identified, including an endolysin and an overlapping bimolecular spanin complex.

### Accession number(s).

The genome sequence of phage Mijalis was deposited under GenBank accession no. KY654690.
